# Effectiveness of Mobile Health Augmented Cardiac Rehabilitation (MCard) on health-related quality of life among post-acute coronary syndrome patients: A randomized controlled trial

**DOI:** 10.12669/pjms.38.3.4724

**Published:** 2022

**Authors:** Aliya Hisam, Zia Ul Haq, Sohail Aziz, Patrick Doherty, Jill Pell

**Affiliations:** 1 Aliya Hisam, MBBS, MPH, FCPS, PhD Public Health. Associate Professor, Department of Community Medicine, Army Medical College, National University of Medical Sciences (NUMS), Rawalpindi, Pakistan; 2 Prof. Zia Ul Haq, MBBS, MPH, PhD. Department of Public Health & Social Sciences, Khyber Medical University, Peshawar, Pakistan; 3 Prof. Sohail Aziz, MBBS, BSc, MRCP, FCPS, M.Phil, FSCH. Consultant Interventional Cardiologist, Armed Forces Institute of Cardiology and National Institute of Heart Diseases (AFIC/NIHD), Rawalpindi, Pakistan; 4 Prof. Patrick Doherty, PhD. Department of Health Sciences, University of York, United Kingdom; 5 Prof. Jill Pell, MBChB, MD, FFPH. Institutes of Health & Wellbeing, University of Glasgow, United Kingdom

**Keywords:** Acute coronary syndrome, cardiac rehabilitation, cardiovascular diseases, health-related quality of life, MacNew QLMI, Mobile health augmented cardiac rehabilitation (MCard), Short form 12

## Abstract

**Objectives:**

To determine the effectiveness of Mobile health augmented Cardiac rehabilitation (MCard) on health-related quality of life (HRQoL) among post-acute coronary syndrome (post-ACS) patients.

**Methods:**

At the Armed Forces Institute of Cardiology (AFIC), a tertiary care hospital in Rawalpindi, Pakistan, a two-arm randomised controlled trial was conducted in which mobile health augmented cardiac rehabilitation (MCard) was developed and implemented on post-ACS patients from January 2019 until March 2021. The trial conforms to the CONSORT statement 2010. The post-ACS patients were randomly allocated (1:1) to an intervention group (received MCard; counselling, empowering with self-monitoring devices, short text messages, in addition to standard post-ACS care) or control group (standard post-ACS care). HRQoL was assessed by generic Short Form-12 and MacNew quality of life myocardial infarction (QLMI) tools. Participants were followed for 24 weeks with data collection and analysis at three time points (baseline, 12 weeks and 24 weeks).

**Results:**

At baseline, 160 patients (80 in each group; mean age 52.66±8.46 years; 126 male, 78.75%) were recruited, of which 121(75.62%) continued and were analysed at 12-weeks and 119(74.37%) at 24-weeks. The mean SF-12 physical component score significantly improved in the MCard group at 12 weeks follow-up (48.93 vs control 43.87, p<.001) and 24 weeks (53.52 vs 46.82 p<.001). The mean SF-12 mental component scores also improved significantly in the MCard group at 12 weeks follow-up (44.84 vs control 41.40, p<.001) and 24 weeks follow-up (48.95 vs 40.12, p<.001). At 12-and 24-week follow-up, all domains of MacNew QLMI (social, emotional, physical and global) were also statistically significant (p<.001) improved in the MCard group, unlike the control group.

**Conclusion:**

MCard is an effective and acceptable intervention at improving all domains of HRQoL. There was an improvement in physical, mental, social, emotional and global domains among the MCard group in comparison to the control group. The addition of MCard programs to post-ACS standard care may improve patient outcomes and reduce the burden on the health care setting.

## INTRODUCTION

Cardiovascular diseases (CVDs) are the major public health problem, claiming the lives of 17.9 million people per year worldwide. In low-and middle-income countries, these non-communicable diseases are causing 82% of premature deaths altogether.^1^ Acute coronary syndrome (ACS) is one of the most common CVDs, which affects about 12 million people annually, with 600,000 of them dying.^2^ These premature deaths can be minimised by using population-wide approaches to mitigate lifestyle risk factors, namely tobacco use, sedentary lifestyle and malnutrition, physical inactivity, and harmful alcohol use. Despite modern cardiovascular early diagnosis and advance medications, these diseases still have high morbidity and mortality.^2^-^4^

ACS has been documented in many studies to substantially impact the sufferer’s health-related quality of life (HRQoL), which is as essential as other clinical outcomes. HRQoL is a multidimensional term that encompasses a person’s physical, emotional, and social well-being that is a well-known indicator of mortality in the general population and mortality and morbidity after ACS diagnosis or related event.^5^ As a secondary preventive measure, CR is a professionally administered programme first introduced in the 1960s and 1970s as a critical tool for stabilising patients following a severe cardiac event (myocardial infarction or cardiac surgery).^6^

The American Heart Association (AHA) has recommended CR, and it has been advised in clinical practice guidelines, but post-ACS patients’ participation in CR programmes is extremely limited and underutilised.^6^-^8^ This is especially true in low-resource areas, like Pakistan, where the epidemic is most severe. The reasons are numerous and include obstacles in the healthcare system, programmes, and at the patient level. The problem lies in CR underutilisation as about 20% or fewer patients enrol in them therefore AHA has also stressed the importance of incorporating newer methods for chronic disease treatment that can be delivered over the phone, the internet, or other forms of communication.^9^,^10^

Health initiatives are nowadays enabled by mobile health (mHealth), in which smartphone applications have shown positive health effects in secondary prevention. During the ongoing COVID-19 crisis, the role of mhealth in health care is becoming increasingly relevant. There is a need to assess the effectiveness of mHealth-based CR in future studies.^11^,^12^

In Pakistan, where the public health system is still underdeveloped,^13^ the CR can significantly improve functional outcomes and quality of life, it is rarely used in clinical settings across the country. CR’s mhealth transition can educate and encourage patients in self-management, physical activity, healthy diet and other lifestyle modifications. This randomised controlled trial aimed to develop and evaluate the effectiveness of Mobile health augmented Cardiac rehabilitation (MCard) at improving HRQoL in post-ACS patients.

## METHODS

At the Armed Forces Institute of Cardiology (AFIC), a tertiary care hospital in Rawalpindi, Pakistan, a two-arm randomised controlled trial was conducted in which mobile health augmented cardiac rehabilitation (MCard) was developed and implemented on post-ACS patients from January 2019 until March 2021. The trial conforms to the CONSORT statement 2010. The CONSORT checklist is attached in Supplementary File 1. Post-ACS patients (ST-elevation myocardial infarction, non-ST elevation myocardial infarction, and unstable angina) admitted to AFIC during the study period were identified and enrolled after applying eligibility criteria. All the participants were given self-monitoring devices (digital blood pressure apparatus, weight machine and pedometer) along with a booklet to record their measurements.

The intervention group received the MCard intervention, a medically supervised cardiac rehabilitation program in addition to standard post-ACS care. The first phase of the MCard included individualised psychotherapy during the hospital stay. The second phase included diurnal mobile texting of standardised messages about healthy lifestyle changes through a specially developed app. The control group received standard post-ACS care. The trial protocol in its entirety has already been published.^14^ The trial is also registered in the Australian New Zealand Clinical Trial Registry (ANZCTR) (ACTRN12619001731189).^15^

Data were collected at three-time points, at baseline, 12 weeks follow-up, and then at 24 weeks follow-up, by a research associate who was blinded to the group status of the enrolled participants.

The primary outcome was HRQoL, which was calculated using a standardised HRQoL short form 12 (SF-12) and MacNew quality of life after myocardial infarction (MacNew QLMI Data were entered and analysed in STATA 14. Categorical data were presented as frequencies and percentages, and the two groups were compared using chi-square tests. For continuous data, means with 95% confidence intervals (95% CI) were presented and, for comparisons, independent sample t-tests were used where appropriate. A p-value of <0.05 was taken as significant.

### Ethical Approval

(Ref: DIR/KMU-EB/MII/000486, Dated: 19-11-2018).

## RESULTS

**Fig.1:**
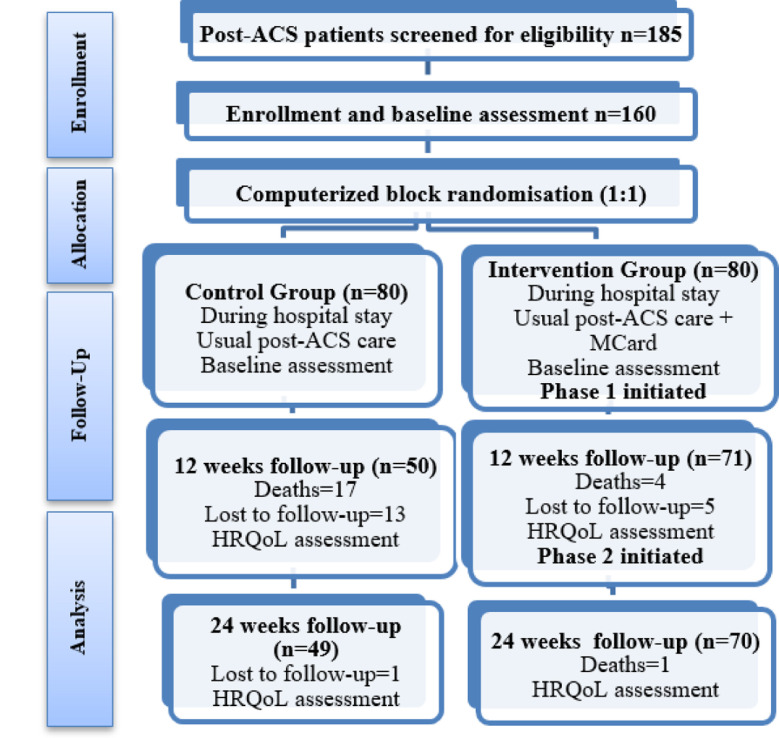
CONSORT Flow chart describing patients presenting post-ACS and eligible for cardiac rehabilitation at Armed Forces Institute of Cardiology, Pakistan.

A total of 185 eligible patients were screened for the study. Twenty-two were not eligible, and three declined to participate. One hundred and sixty post-ACS patients were included and evenly randomised in a 1:1 ratio into two groups of 80 (control and intervention). At 12 weeks follow-up, 121 (75.62%) were analysed as 18 were lost to follow up (control: 13, intervention; 5) and 21 died (control: 17, intervention; 4). An additional one was lost to follow-up (control), and one died (intervention) at 24 weeks, leaving 119 (74.37%) with complete data. (Fig.1)

The participants’ mean age at baseline was 52.66 ± 8.46 years. Overall, predominantly men were enrolled (n=126, 78.75%) as compared to females (n=34, 21.25%). Punjabi ethnicity was the majority (n= 119, 74.38%), followed by Pashtun (n=21, 13.13%) (Table-I).

**Table I:** Sociodemographic and clinical characteristics of the control and intervention groups.

	Overall (n=160)	Control group (n=80)	Intervention group (n=80)	p-value

	(%)	n (%)	n (%)	
***Age Mean (SD)***	52.66 (8.46)	51.64 (7.13)	53.70 (9.56)	0.124
***Gender***				
Male	126 (78.75)	75 (93.75)	51 (63.75)	<0.001
Female	34 (21.25)	05 (06.25)	29 (36.25)	
***Ethnicity***				
Punjabi	119 (74.38)	60 (75.00)	59 (73.75)	0.368
Pashtun	21 (13.13)	08 (10.00)	13 (16.25)	
Others	20 (12.50)	12 (15.00)	08 (10.00)	
***Education***				
No formal education	49 (30.63)	24 (30.00)	25 (31.25)	0.881
Primary	13 (08.13)	08 (10.00)	05 (06.25)	
Middle	16 (10.00)	07 (08.75)	09 (11.25)	
Secondary	28 (17.50)	13 (16.25)	15 (18.75)	
Higher >10	54 (33.75)	28 (35.00)	26 (32.50)	
***Employment***				
Unemployed	86 (53.75)	39 (48.75)	47 (58.75)	0.205
Employed	74 (46.25)	41 (51.25)	33 (41.25)	
***Income per month***				
mean (SD)	40583.75 (21169.78)	37667.50 (17338.28)	43500 (24169.23)	0.081
***ACS type***				
ST elevation	110 (68.75)	54 (67.50)	56 (70.00)	0.733
Non-ST elevation	29 (18.13)	12 (15.00)	17 (21.25)	0.305
Unstable angina	21 (13.13)	14 (17.50)	07 (08.75)	0.101
***Management***				
Revascularization	107 (66.88)	48 (60.00)	59 (73.75)	0.065
Thrombo. therapy	19 (11.88)	12 (15.00)	07 (08.75)	0.222
CABG	13 (08.13)	09 (11.25)	04 (05.00)	0.148
***Comorbidities***				
Hypertension	82 (51.25)	35 (43.75)	47 (58.75)	0.058
Diabetes	68 (42.50)	33 (41.25)	35 (43.75)	0.749
Hyperlipidemia	08 (05.00)	06 (07.50)	02 (02.50)	0.147
Cerebrovas. event	03 (01.88)	03 (03.75)	-	0.080
Others	06 (03.75)	02 (02.50)	04 (05.00)	0.405

**Fig.2:**
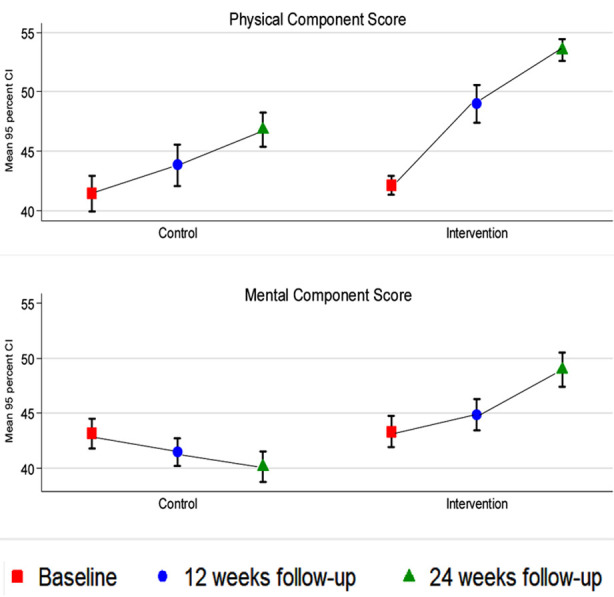
Comparison of HRQOL domains mean score changes at baseline, 12 weeks and 24 weeks among the two groups.

The mean physical component scores for the control and intervention groups were 41.67, 95% CI 40.62, 42.73 vs 41.78, 95% CI 40.96, 42.59 at baseline (p-value=0.879), 43.87, 95% CI 42.17, 45.58, vs 48.93, 95% CI, 47.35, 50.50 at 12 weeks follow-up (p-value<.001), and 46.82, 95% CI 45.37, 48.26 vs 53.52, 95% CI 52.57, 54.46 at 24 weeks follow-up (p-value<.001). The mean mental component scores for the control and intervention groups were 43.13, 95% CI 41.97, 44.29 vs 43.36, 95% CI 41.99, 44.73 at baseline (p-value=0.801), 41.40, 95% CI 40.18, 42.62 vs 44.84, 95% CI 43.42, 46.26, at 12 weeks follow-up (p-value<.001), and 40.12, 95% CI 38.71, 41.53 vs 48.95, 95% CI 47.42, 50.49, at 24 weeks follow-up (p-value<.001). Consistent with the two component scores, almost all the domains of SF-12 (other than RP and RE domains) showed significant increase among the intervention group, in contrast to the control group, at both follow-up periods (Table-II, Fig.2).

**Table II:** Health-related quality of life, assessed by Short Form 12, at baseline and follow-up.

	Baseline		12 weeks		24 weeks	

	Mean (95% CI)	p-value	Mean (95% CI)	p-value	Mean (95% CI)	p-value
***Physical function***						
Control	38.93 (36.91,40.94)	0.467	42.26 (39.97, 44.55)	<.001	45.65 (43.25, 48.05)	<.001
Intervention	39.80 (38.51,41.09)		48.96 (46.61, 51.31)		54.81 (53.48, 56.13)	
***Role physical***						
Control	39.95 (38.71, 41.19)	0.495	40.87 (39.52, 42.22)	0.311	41.40 (40.26, 42.53)	0.111
Intervention	40.53 (39.37, 41.69)		41.84 (40.55, 43.14)		43.43 (41.47, 45.40)	
***Bodily pain***						
Control	41.71 (39.88, 43.55)	0.460	41.49 (39.42, 43.56)	<.001	43.92 (41.73, 46.11)	<.001
Intervention	42.62 (41.03, 44.21)		48.45 (46.65, 50.25)		53.34 (51.89, 54.80)	
***General Health***						
Control	44.91 (42.52, 47.31)	0.122	44.69 (42.26, 47.11)	<.001	47.14 (45.37, 48.91)	<.001
Intervention	47.22 (45.48, 48.96)		51.41 (49.69, 53.14)		56.01 (54.94, 57.08)	
***Vitality***						
Control	49.55 (47.65, 51.46)	0.075	49.46 (47.77, 51.15)	0.003	48.26 (46.45, 50.07)	<.001
Intervention	51.77 (50.21, 53.32)		53.22 (51.47, 54.96)		59.46 (58.27, 60.65)	
***Social functioning***						
Control	40.22 (39.07, 41.37)	0.789	40.35 (38.91, 41.80)	0.460	40.56 (38.96, 42.15)	<.001
Intervention	40.44 (39.26, 41.62)		41.24 (39.51, 42.96)		48.64 (46.97, 50.31)	
***Role emotional***						
Control	35.68 (33.86, 37.50)	0.914	35.90 (34.22, 37.59)	0.281	36.55 (35.18, 37.91)	0.111
Intervention	35.81 (34.28. 37.34)		37.17 (35.60, 38.74)		39.05 (36.63, 41.47)	
***Mental health***						
Control	44.77 (43.22, 46.33)	0.387	42.63 (40.70, 44.57)	<.001	42.19 (40.06, 44.33)	<.001
Intervention	45.78 (44.07, 47.48)		51.36 (49.59, 53.13)		55.27 (53.98, 56.56)	
***Physical component score***						
Control	41.67 (40.62, 42.73)	0.879	43.87 (42.17, 45.58)	<.001	46.82 (45.37, 48.26)	<.001
Intervention	41.78 (40.96, 42.59)		48.93 (47.35, 50.50)		53.52 (52.57, 54.46)	
***Mental component score***						
Control	43.13 (41.97, 44.29)	0.801	41.40 (40.18, 42.62)	<.001	40.12 (38.71, 41.53)	<.001
Intervention	43.36 (41.99, 44.73)		44.84 (43.42, 46.26)		48.95 (47.42, 50.49)	

**Fig.3:**
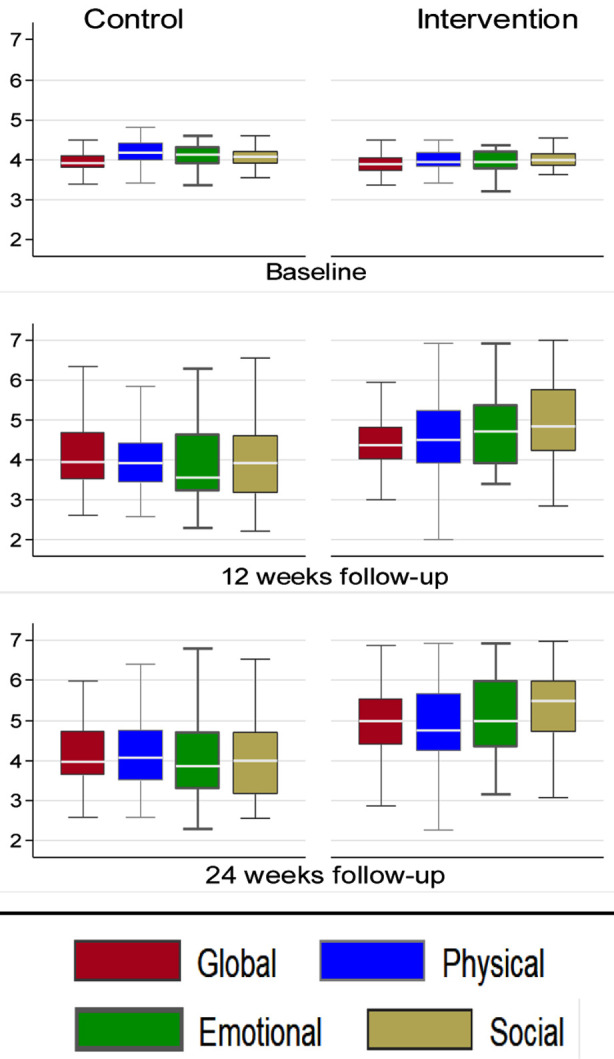
Comparison of MacNew QLMI mean scores at baseline, 12 weeks and 24 weeks follow-ups among the two groups.

At 12- and 24-weeks follow-up, all the domains of MacNew QLMI showed statistically significant increase among the MCard vs control group in their mean scores. At 12 weeks follow-up, the social MacNew QLMI was 4.05, 95% CI: 3.82, 4.29 among control vs 4.96, 95% CI: 4.75, 5.17, p-value<.001 intervention. This was followed by the emotional domain, 3.92, 95% CI: 3.68, 4.17 vs 4.73, 95% CI: 4.51, 4.94, p-value<.001, then physical domain 4.05, 95% CI: 3.85, 4.25 vs 4.55, 95% CI: 4.32, 4.78, p-value=.001 and lastly global domain, 4.10, 95% CI: 3.91, 4.29 vs 4.49, 95% CI: 4.32, 4.66, p-value=.002, respectively. At 24 weeks follow-up, the social MacNew QLMI mean score was increased significantly among the intervention groups vs controls (5.28, 95% CI: 5.07, 5.50, p-value<.001 vs 4.08, 95% CI: 3.84, 4.31, p-value<.001). There was also an increased scored of emotional domain, (5.22, 95% CI: 4.99, 5.45 vs 4.04, 95% CI: 3.81, 4.27, p-value<.001), then physical domain, (4.80, 95% CI: 4.58, 5.02 vs 4.20, 95% CI: 3.99, 4.40, p-value<.001) and in global domain (4.96, 95% CI: 4.76, 5.16 vs 4.10, 95% CI: 3.93, 4.28, p-value=0.001) (Table-III, Fig.3).

**Table III:** Myocardial infarction specific MacNew QLMI among the control and intervention groups.

	Baseline		12 weeks		24 weeks	

	Mean (95% CI)	p-value	Mean (95% CI)	p-value	Mean (95% CI)	p-value
***MacNew Global***						
Control	3.94 (3.87, 4.01)	0.141	4.10 (3.91, 4.29)	0.002	4.10 (3.93, 4.28)	<0.001
Intervention	3.87 (3.81, 3.93)		4.49 (4.32, 4.66)		4.96 (4.76, 5.16)	
***MacNew Physical***						
Control	4.07 (3.98, 4.17)	0.079	4.05 (3.85, 4.25)	0.001	4.20 (3.99, 4.40)	<0.001
Intervention	3.98 (3.92, 4.03)		4.55 (4.32, 4.78)		4.80 (4.58, 5.02)	
***MacNew Emotional***						
Control	3.77 (3.71, 3.82)	0.565	3.92 (3.68, 4.17)	<0.001	4.04 (3.81, 4.27)	<0.001
Intervention	3.79 (3.73, 3.85)		4.73 (4.51, 4.94)		5.22 (4.99, 5.45)	
***MacNew Social***						
Control	4.03 (3.98, 4.09)	0.810	4.05 (3.82, 4.29)	<0.001	4.08 (3.84, 4.31)	<0.001
Intervention	4.02 (3.97, 4.08)		4.96 (4.75, 5.17)		5.28 (5.07, 5.50)	

## DISCUSSION

This trial used mhealth to encounter the underutilisation of CR and provided evidence-based results that the two-component score (PCS and MCS) and the eight domains measured by SF-12 were significantly better among the intervention group than the control group. Similarly, a disease-specific measure of HRQoL, the MacNew QLMI, also demonstrated improvements in the intervention group at 12- and 24-weeks follow-up in all domains (social, emotional, physical and global) compared with the baseline.

The average age of the enrolled participants was 71.1±10 years, which is higher than the average age of the participants in this sample (52.69 ± 8.47 years), most likely due to the Pakistani ACS’s population’s low median age. However, 160 post-ACS patients were included, which is similar to the number achieved by Saadi et al.^10^ The majority (73.5%) of the study population were male, similar to this study (80 per cent). A mhealth based study^11^ on a total of 34 participants at 12 months follow-up period concluded that HRQoL was significantly improved in terms of both PCS and MCS using SF-36, similar to the result observed in this analysis showed improvements in two-component scores and eight HRQoL domains as measured by SF-12. At 24 weeks, the mean PCS was 49 (95% CI: 48, 51), while the mean MCS was 52. (95% CI: 50, 54). These findings are comparable to this research, in which the mean PCS and MCS in the intervention group were 53.62 (95 percent CI: 52.73, 54.52) and 48.87 (95 percent CI: 47.42, 50.33) at 24 weeks, respectively. According to the findings of one randomised trial, a home-based CR programme with monthly reinforcements has no additional long-term functional benefit over a regular, 4-week outpatient CR programme.^16^ This study contradicts this because it was a home-based clinical trial in which reinforcements were provided through mobile texting. The advanced mhealth technology used in this study design may be the reason for the discrepancy but needs further multicenter trial to validate.^16^

Another study concluded that all the MacNew QLMI domains (physical, emotional, social, overall) showed a substantial difference in their mean scores (P<0.001, P<0.001, P=0.003, and P<0.001 respectively)^17^ which are consistent with this study’s results in which improved MacNew QLMI domains were observed as well. A systematic review also concluded that those receiving CR have shown improved HRQOL domains (global, physical, emotional and social) compared to the control group.^18^ Even though the changes in HRQOL were slight, they nevertheless represent general improvements in effectiveness compared with standard treatment. This is similar to this study results as all MacNew QLMI parameters have improved in the MCard group. Also, the MCard is low cost as the application has been made, and it will be only the cost of text messages to bear.

### Limitations of the study

Firstly, it was a single-centre study; a larger study in future can be planned as multi-centre trials to see the effect in a larger patient population across different geographical sites. Secondly, different subgroups of patients who completed the MCard intervention may benefit differently from the intervention. This research had key strengths, one of which was that all of the analyses were done based on intention to treat. Furthermore, extended follow-up was included to investigate whether benefits achieved at 12 months persisted after that.

Our findings do not undermine the significance of conventional CR; alternately, they demonstrate the value of mhealth in CR by increasing CR utilisation, especially for patients who may not otherwise join in CR due to various circumstances. Since behaviour change is a gradual process, any long-term effects of MCard CR can be measured after 2–5 years, thereby addressing a gap in current knowledge. Expanding MCard services around the country could help many post-ACS patients improve their physical and mental well-being and decrease the country’s non-communicable disease burden.

## CONCLUSION

The MCard intervention is acceptable in a developing country hospital setting and has shown significant improvement in all domains of generic and disease-specific HRQoL compared to the control group. There was an improvement in the physical, mental, social, emotional and global domains among the MCard group compared to the control group. Hence, our MCard program may be adapted and added to the secondary prevention of post-ACS in all tertiary care hospitals. Our findings suggest this may also improve patient outcomes and reduce the burden on the health care setting, including outpatient physicians, in the longer run.

### Authors’ contributions:

**AH:** Conceived and designed trial design, data acquisition and analysis, drafting work. Responsible and accountable for the accuracy or integrity of the work.

**ZUH:** Conception, design of the work and manuscript revision critically.

**SA:** Design of the work and conduct of the trial design, review of the final manuscript.

**PD, JP:** Review and final approval of the manuscript to be published.
